# Minimal clinically important difference for improvement in six-minute walk test in persons with knee osteoarthritis after total knee arthroplasty

**DOI:** 10.1186/s12891-022-05262-4

**Published:** 2022-03-31

**Authors:** Lauren K. King, Gillian A. Hawker, Ian Stanaitis, Linda Woodhouse, C. Allyson Jones, Esther J. Waugh

**Affiliations:** 1 Department of Medicine, University of Toronto, Toronto, ON M5S 3H2 Canada; 2 Women’s College Research Institute, Women’s College Hospital, 6307 - 76 Grenville Street, 6th Floor, Toronto, ON M5S 1B2 Canada; 3 Department of Physical Therapy, Faculty of Rehabilitation Medicine, University of Alberta, Edmonton, AB T6G 2G4 Canada; 4 Department of Physical Therapy, University of Toronto, Toronto, ON M5G 1V7 Canada

**Keywords:** Minimal clinically important difference, Knee osteoarthritis, Total knee arthroplasty, Six-minute walk test

## Abstract

**Background:**

The interpretability of the six-minute walk test (6MWT) in individuals with knee osteoarthritis (OA) is unclear. We aimed to determine the minimal clinically important difference (MCID) for improvement in 6MWT in persons with knee OA at 12 months after total knee arthroplasty (TKA), and if it differed by baseline walking ability.

**Methods:**

Participants with knee OA were assessed 1 month pre- and 12 months post-TKA, including completion of 6MWT. At 12 months, participant-perceived change in walking ability was assessed on an 8-point Likert scale ranging from “extremely worse” to “extremely better”. Using logistic regression, ROC curves examined the ability of change in 6MWT distance to discriminate those who perceived walking was improved. MCID was selected overall and then by quartile of baseline 6MWT distance using the Youden method.

**Results:**

Two hundred seventy-eight participants were included: mean age 67 years (SD 8.5), 65.5% female, mean pre-TKA 6MWT distance 323.1 (SD 104.7) m, and mean 12-mo 6MWT distance 396.0 (SD 111.9) m. The overall MCID was 74.3 m (AUC 0.65). Acceptable model discrimination (AUC > 0.70) was achieved for individuals in the lowest quartiles of baseline 6MWT distance: Quartile 1: MCID 88.63 m (AUC 0.73); Quartile 2: MCID 84.47 m (AUC 0.72).

**Conclusions:**

In persons with knee OA 12 months post-TKA, 6MWT MCID is dependent on baseline walking ability. Poor model discrimination for those in the highest (best) quartiles of baseline walking ability raise questions about 6MWT use across the full spectrum of walking ability. Further research is needed to better understand use of 6MWT as a performance-based measure of physical function for persons with knee OA.

## Background

Improvement in physical function is one of the primary reasons that patients with chronic, painful, and disabling knee osteoarthritis (OA) seek care [1, 2]. Physical function is considered a mandatory outcome in all knee OA clinical trials [3], and can be assessed through patient-reported or performance-based measures. The six-minute walk test (6MWT) is in the Osteoarthritis Research Society International (OARSI) recommended set of performance-based tests of physical function in patients with knee OA as a test of submaximal aerobic capacity and ability to walk over long distances [4]. It has been shown to predict 30-min walk test performance in persons with knee OA [5]. The 6MWT, however, was initially developed to evaluate functional capacity in frail older adults [6]. While its use has expanded to a variety of chronic diseases, including predicting morbidity and mortality in persons with cardiovascular and respiratory diseases [7–9], the interpretability of 6MWT in individuals with knee OA remains unclear, including what change in 6MWT constitutes a meaningful improvement in walking ability.

The minimal clinically important difference (MCID) identifies the smallest amount of change in an outcome that is meaningful, and is considered best assessed using an anchor-based approach linking to patients’ perceptions of important change [10–12]. In persons with knee OA, Naylor et al. [13] reported an anchor-based MCID for improvement in 6MWT at 6 months after total knee arthroplasty (TKA) of 26.0 m using Youden method [14], with the patient report of walking “slighter better” or greater as the external anchor to designate meaningful improvement. When they calculated MCID at different thresholds of change (“moderate” and “much better”) that may better reflect important clinical improvement after TKA, model performance was suboptimal. While they also calculated a MCID using two distribution-based methods, reporting values of 37.9 m and 55.4 m [13], these estimates do not incorporate patients’ judgement of improvement [15] and therefore their meaning is unclear. In two other studies of patients with knee OA prior to total joint arthroplasty, minimal detectable change at the 90% confidence level was calculated as 66.3 m [16] and 61.3 m [17] (the latter also included individuals with hip OA). Several knowledge gaps remain. There is currently no estimate of MCID at 12 months after TKA, a common follow-up point in knee OA studies. It also remains unclear how a 6MWT MCID might vary based on different patient attributes, in particular, baseline walking ability. MCID has been increasingly shown to be context specific, rather than a fixed value [18, 19]. In individuals with chronic pain, baseline pain level has been shown to be the main cause of variation in MCID between studies [20].

Therefore, the purpose of this study was to calculate an anchor-based MCID for 6MWT in individuals with knee OA at 12 months after primary, elective TKA, and whether it differs by pre-operative 6MWT walking distance. Our findings, however, raised further questions about the use of 6MWT as an outcome in persons with knee OA.

## Methods

### Design and study sample

This was a secondary analysis of a prospective cohort study. Our study population comprised a convenience sample of participants from the BEST-Knee cohort study [21, 22], who were assessed at the Edmonton, Canada, provincial hip and knee clinic site. The study recruited adults aged 30 years and older with primary knee OA referred for consultation regarding elective primary unilateral TKA between October 27, 2014 and September 30, 2016. Study participants enrolled at the Edmonton centre scheduled for TKA were invited to complete the 6MWT, aiming for a target of 300 participants based on study funding and feasibility. All participants were required to be able to read and comprehend English to provide written informed consent. Those undergoing revision or bilateral TKA, or with a diagnosis of inflammatory arthritis were excluded. Individuals with prior TKA on the contralateral knee were eligible to participate.

### Assessments

#### Participant characteristics

Participants completed standardized questionnaires 1 month prior to and 12 months after TKA. The baseline questionnaire assessed demographic characteristics, including participant age, gender, level of education (post-secondary education vs. no post-secondary education), and current smoking status (yes/no). Patient-reported knee OA pain severity was assessed using the Western Ontario and McMaster Universities Osteoarthritis Index (WOMAC) pain subscale, where a higher score indicates worse pain [23, 24]. Knee OA-related physical function was assessed using the Knee injury and Osteoarthritis Outcome Score physical function short form (KOOS-PS), coded such that a higher score indicates worse disability [25].

Comorbidities were assessed by having participants indicate yes/no from a list of common chronic conditions. The total number of comorbid conditions was summed and categorized as 0, 1, 2, or ≥ 3. Participants were asked to indicate on a homunculus the number of lower extremity joints that had been troublesome (painful, aching, swollen or stiff) on most days of the past 3 months. Number of troublesome joints was summed. The eight-item Patient Health Questionnaire Depression Scale (PHQ-8) [26] was used to assess depressive symptoms. Body mass index (BMI) was calculated using participants’ height and weight obtained from clinic records.

#### 6MWT

6MWT was performed within 1 month prior to TKA and 12 months after TKA. 6MWT was assessed with or without gait aids on a 20 m measured indoor loop. Rests were permitted but time was not stopped.

#### Perceived change in walking ability (anchor question)

At 12 months after TKA, participants’ perceived change in walking ability was assessed on an 8-point Likert scale: *“*Compared to before your surgery, has there been any changes in your walking?”. Response options were: “Extremely worse”, “a lot worse”, “somewhat worse”, “unchanged”, “a little better”, “somewhat better”, “a lot better”, or “extremely better”.

### Statistical analyses

Distributions of all continuous variables were assessed for normality. WOMAC pain scores were transformed to a score from 0 to 100. Participant characteristics were summarized using frequencies, means and standard deviations (SD) or medians and interquartile ranges (IQR), as appropriate. Pre-TKA and 12-month 6MWT distances were summarized overall and by quartile of pre-TKA 6MWT distance. 6MWT change distance was calculated by subtracting pre-TKA distance from 12-month distance. The mean change in 6MWT by pre-TKA 6MWT quartile was compared by ANOVA test.

Assessment of MCID followed an anchor-based approach [10]. This approach, incorporating patients’ perception of change, has been used when calculating MCID for 6MWT across a range of diseases [27]. Spearman rank correlations examined correlations between the patient-reported perceived walking improvement (anchor question) responses on the 8-point scale and change in 6MWT distance. Prior authors have suggested that correlation coefficients between the patient-based anchor and outcome ≥0.3 [28] indicate an appropriate anchor is selected. The MCID for improvement was calculated using receiver-operating characteristic (ROC) curves [15]. Given the prior validated threshold for important change after TKA of “a good deal better” [29], we used the patient-reported criterion of walking “a lot better” or “extremely better” to define clinically important improvement. Participants were thus dichotomized as *improved* if they indicated change in walking was “a lot better”, or “extremely better”, and *non-improved* if they indicated walking as “somewhat better”, “a little better”, “unchanged”, “somewhat worse”, “a lot worse” or “extremely worse”. The logistic regression model AUC assessed the ability of 6MWT distance to discriminate those who were *improved* vs. *non-improved*. The Youden method [14] was used to select a MCID that maximized both sensitivity and specificity. To understand the effect of baseline walking ability on MCID threshold, the analysis was repeated for each quartile of baseline 6MWT test distance. Baseline walking ability takes into account a multitude of factors that can influence walking ability in persons with knee OA undergoing TKA [30].

All statistical analyses were performed using SAS Studio Version 3.8 (SAS Institute Inc., Cary, NC). Statistical significance was considered met at a two-sided *p*-value of 0.05.

### Ethics approval

The study was approved by the Research Ethics Boards of the University of Alberta (PRO-00051108) and Women’s College Hospital (REB 2014-0092) at the University of Toronto. All participants provided informed written consent. All methods were carried out in accordance with relevant guidelines and regulations.

## Results

### Participant characteristics pre-TKA

Three hundred one participants completed the 6MWT prior to TKA; of these, 278 (92.3%) had complete data for 6MWT at 12 months and were included in our analyses. Mean age was 67 years (SD 8.5) and 65.5% were female. Mean pre-TKA WOMAC pain was 57.2 (SD 16.5)/100, mean pre-TKA KOOS-PS was 52.9 (SD 16.2)/100 and mean pre-TKA 6MWT distance 323.1 (SD 104.7) m. At 12 months post TKA, mean WOMAC pain was 14.2 (SD 15.3)/100, KOOS-PS was 22.9 (SD 15.8)/100, mean 12-month 6MWT distance was 396.0 (SD 111.9) m, and mean improvement in 6MWT at 12 months was 72.9 m (SD 91.0). The majority of participants had other co-existing condition (1 condition: 36.3%, 2 conditions: 20.9, ≥3 conditions: 9.0%). The most common comorbidities were back pain (54.4%), hypertension (48.9%), cardiovascular disease (14.5%), depression (22.0%), gastrointestinal disease (13.0%), respiratory disease (8.9%), and cancer (5.6%). Participant characteristics are shown in Table 1.

**Table 1:** Baseline characteristics of participants (*n* = 278)

Characteristic	
*Pre-TKA demographics*	
Mean age (years) (SD)	67.10 (8.52)/278
Female – no. (%)	182 (65.47)/278
Post-secondary education – no. (%)	155 (56.57)/274
Current smoking – no. (%)	17 (6.16)/276
Body mass index (kg/m^2^) (SD)	32.19 (6.08)/278
*Pre-TKA knee OA characteristics*	
Mean WOMAC pain (0-100) (SD)	57.22 (16.53)/278
Mean KOOS physical function short form (0-100) (SD)	52.92 (16.25)/278
*Pre-TKA comorbidities*	
Mean PHQ-8 depressive symptoms (0-24) (SD)	6.76 (5.72)
Median total number troublesome joints (including index joint) (IQR)	2 (1, 3)
Number of comorbidities^a^ – no. (%)	
0	94 (33.814)/278
1	101 (36.33)/278
2	58 (20.86)/278
≥ 3	25 (8.99)/278

*TKA* Total knee arthroplasty, *WOMAC* Western Ontario and McMaster Universities Osteoarthritis Index, *KOOS* Knee injury and osteoarthritis outcome score, *PHQ-8* Patient health questionnaire - 8

^a^Not including depression and number of troublesome joints

Participants in lower quartiles of pre-TKA 6MWT distance had greater change in 6MWT at 12-month after TKA (*p* < 0.001) (Table 2).

**Table 2:** Baseline and 12-month 6MWT distances, overall and by quartile of baseline 6MWT distance

	Overall (***n*** = 278)	Quartile 1 (***n*** = 70)	Quartile 2 (***n*** = 69)	Quartile 3 (***n*** = 70)	Quartile 4 (***n*** = 69)
Quartile cut-offs (m)	–	< 255.08	255.08 to 336.26	336.26 to 391.67	> 391.67
Pre-TKA 6MWT (m) (SD)	323.09 (104.66)	185.29 (155.01)	296.85 (23.45)	363.68 (16.44)	447.97 (54.42)
12-month 6MWT (m) (SD)	395.96 (111.87)	301.33 (107.14)	383.94 (78.28)	421.05 (72.00)	478.53 (104.95)
Change 6MWT (m) (SD)	72.86 (90.98)	116.04 (108.90)	87.09 (78.18)	57.37 (70.46)	30.56 (79.69)

*6MWT* Six-minute walk test, *TKA* Total knee arthroplasty

**Fig. 1:**
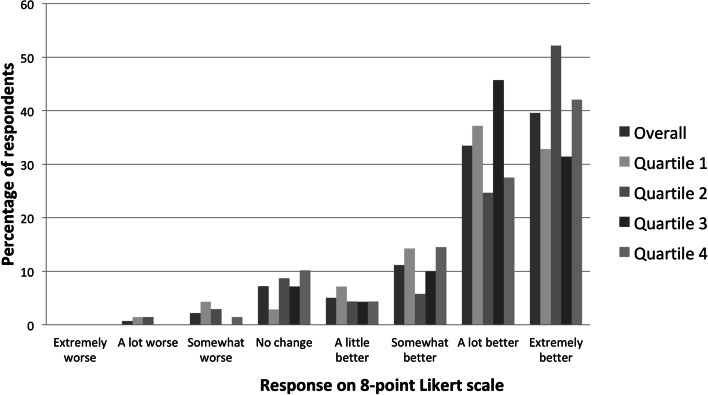
Perceived improvement in walking ability, overall and by quartile of baseline 6MWT distance

Overall, most participants reported that their walking ability improved at 12 months after TKA. Pattern of responses to perceived change in walking ability at 12 months after TKA, overall and by baseline quartile of 6WMT distance, are shown in Fig. 1.

**Fig. 2:**
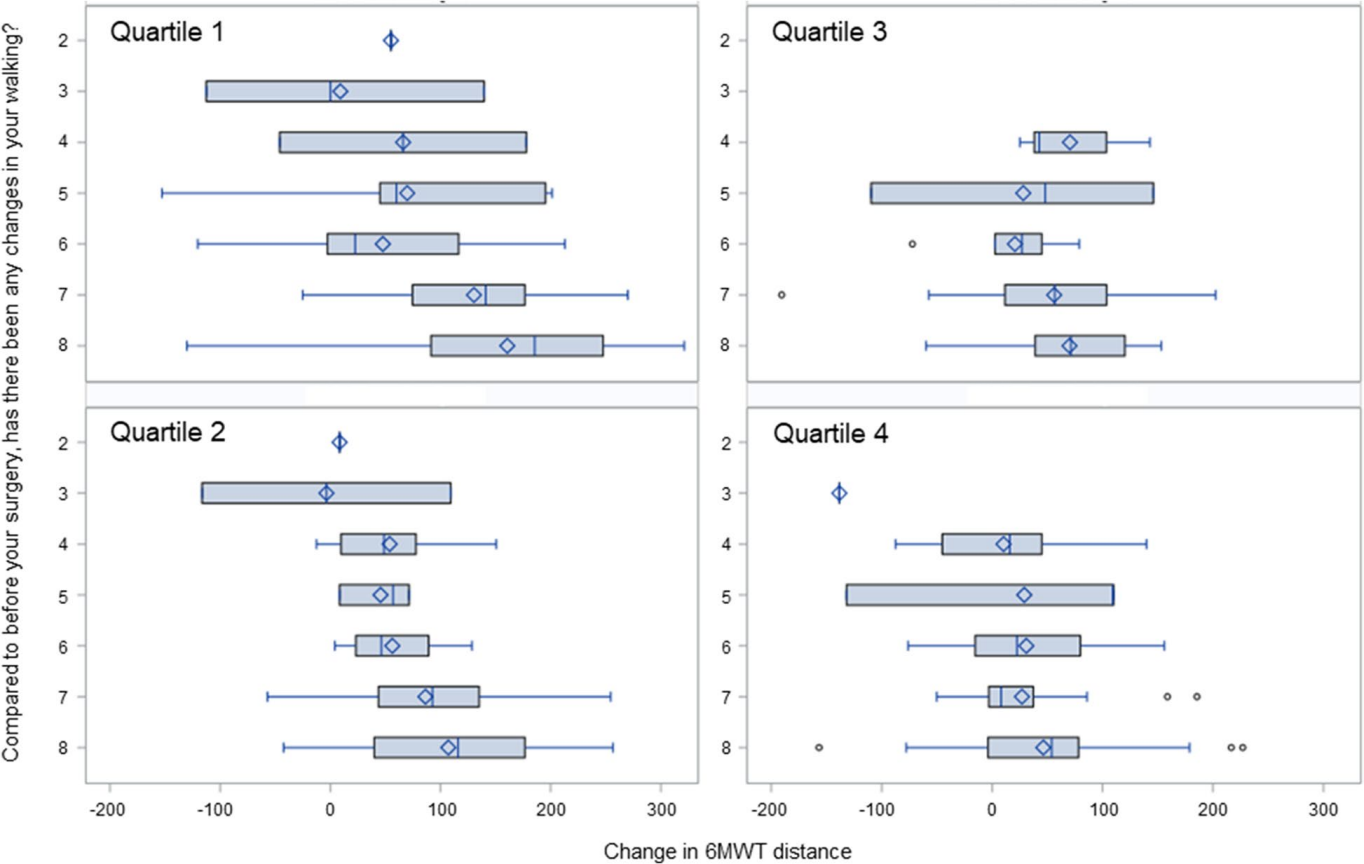
Change in 6MWT distance at 12 months after TKA by perceived change in walking

There was variability in measured change in 6MWT distance for each level of perceived change in walking (Fig. 2). For baseline 6MWT quartiles 1 and 2, generally those who perceived greater improvement in walking ability at 12 months had greater improvement in their 6MWT distance, but this pattern was not observed for quartiles 3 and 4 (Fig. 2).

### Correlation between change in 6WMT and anchor scale

Overall, the correlation between the anchor scale (perceived change) responses and change in 6MWT distance was 0.23 (*p* < 0.001). Correlations varied by quartile of baseline 6MWT distance and were moderate in individuals in the lowest two quartiles (Quartile 1 *r* = 0.40, *p* < 0.001; Quartile 2 *r* = 0.33, *p* = 0.006; Quartile 3 *r* = 0.15, *p* = 0.21; Quartile 4 *r* = 0.16, *p* = 0.18).

### MCID thresholds

**Fig. 3:**
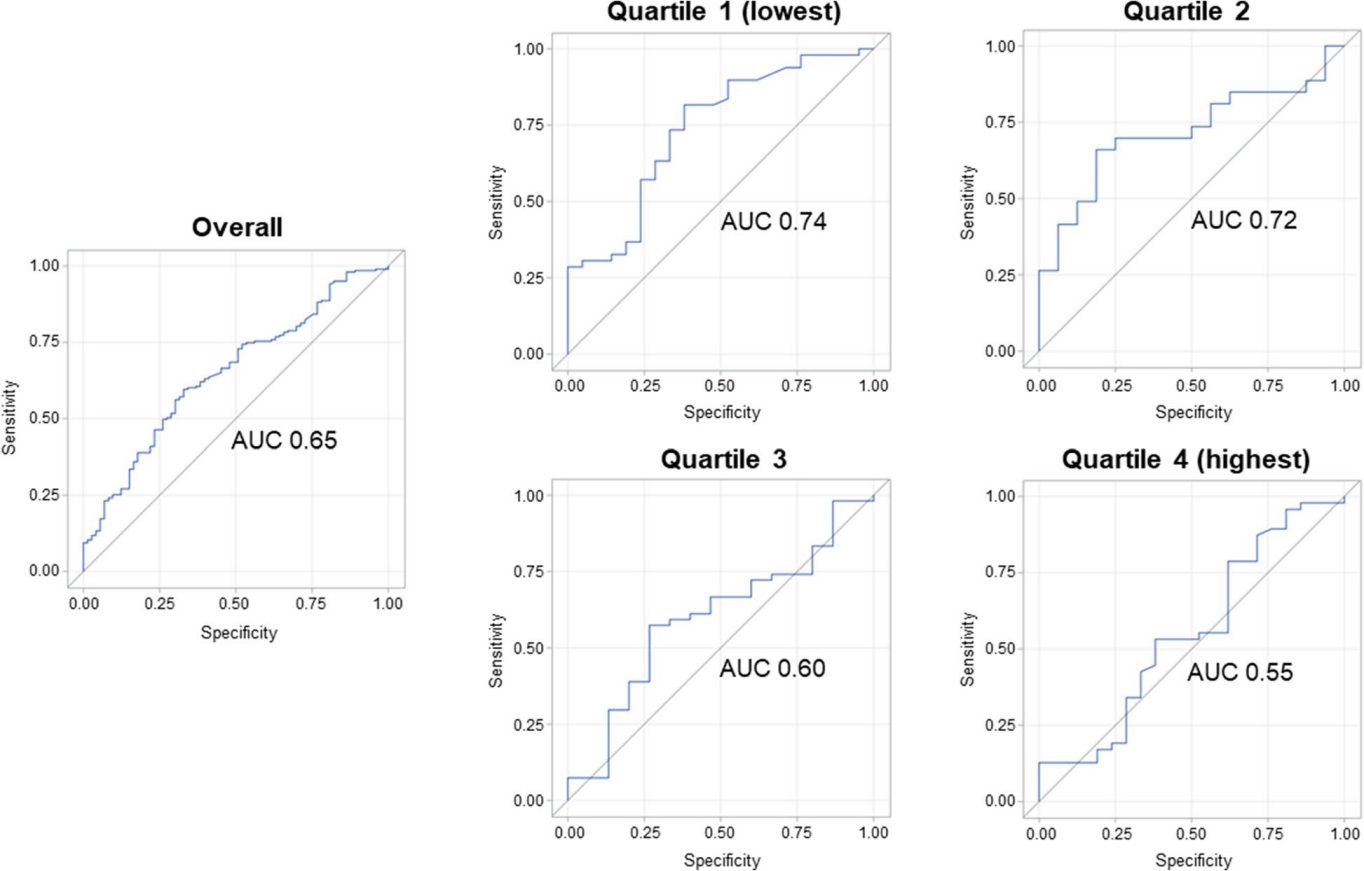
ROC curves showing ability of change 6MWT distance to discriminate those whose walking ability was *improved* vs. *non-improved*

Overall, we identified a MCID threshold of 74.36 m, selected based on the highest J index. This had a sensitivity 0.60, specificity 0.67, and AUC 0.65 for classifying individuals as *improved* versus *non-improved*. The MCID calculated in each quartile of baseline 6MWT distance showed variation; MCID was larger in the lowest quartiles, compared to the overall sample, indicating that a greater change was needed to be considered meaningful. We calculated a MCID for quartile 1 of 88.63 m (AUC 0.74), and quartile 2 of 84.47 m (AUC 0.72). Discrimination was poor in quartile 3 and 4, and thus MCIDs were not calculated (Table 3). ROC curves are shown in Fig. 3.

**Table 3:** Calculated MCID, overall and by quartile of baseline 6MWT distance

	Overall (***n*** = 278)	Quartile 1 (***n*** = 70)	Quartile 2 (***n*** = 69)	Quartile 3 (***n*** = 70)	Quartile 4 (***n*** = 69)
MCID (m)	74.36	88.63	84.47	–	–
Sensitivity	0.60	0.82	0.66	–	–
Specificity	0.67	0.62	0.81	–	–
AUC	0.65	0.74	0.72	0.60	0.55

*MCID* Minimal clinically important difference, *6MWT* Six-minute walk test, *AUC* Area under curve

## Discussion

In a cohort of individuals with knee OA, using an anchor-based approach, we sought to calculate a MCID for improvement in 6MWT at 12 months after TKA overall and by quartile of baseline 6MWT distance. Our goal was to add to the understanding of the interpretability of 6MWT in persons undergoing TKA for knee OA and how MCID might change by baseline walking ability. We found that change in 6MWT distance could only adequately discriminate those who reported their walking was meaningfully *improved* vs *non-improve*d for individuals in the lowest quartiles of baseline walking ability, that is, those prior to TKA who recorded a 6MWT ≤336 m. For these individuals, we estimate a MCID of 88.6 m (quartile 1) to 84.5 m (quartile 2). On average, this represents being able to walk approximately an additional city block [31] in 6 min.

While we calculated an overall MCID value of 74.3 m, given poor model discrimination (AUC 0.65), we do not feel this is a useful threshold for use in clinical research. Naylor et al. also found that model discrimination was suboptimal when calculating MCID at 6 months after TKA [13] at the comparable patient-reported threshold of “much better” to designate meaningful improvement (AUC 0.65). Their study assessed an anchor-based MCID at three different levels of patient-reported improvement in walking. Model performance was best using the threshold of “slightly better” or greater, with AUC 0.72 and both specificity and sensitivity greater than 50%, resulting in their proposed anchor-based MCID at 6 months after TKA of 26.0 m. However, applicability of this is limited given it is unlikely that walking “slightly better” would reflect meaningful improvement to patients after TKA [29]. Thus, determining a MCID using an anchor-based approach must importantly consider, a priori, the appropriate choice of anchor.

The unexpected yet important finding of our study was the difference in ability to classify individuals as *improved* vs *non-improved* between the lower and upper quartile baseline 6MWT walking ability. While we could calculate MCID for those in the lowest quartiles, we found that model discrimination was poor for quartiles 3 and 4 and we felt calculating MCID values would not be meaningful and we have thus not proposed MCIDs for the upper quartiles. Individuals in the upper quartiles showed relatively modest change in their 6MWT distance at 12 months, with high variability, and their change in 6MWT was not correlated with our anchor scale. This raises further questions as to *why.* Potential explanations include, first, lack of specificity of our anchor question. A more specific question relating to submaximal walking ability (e.g., change in ability to walk faster over medium-to-long distances) may have better captured patient’s perceptions of change corresponding to 6MWT. Second, this could reflect the multitude of other factors that might influence an individual’s perception of change, including recall bias. In individuals with chronic pain, perception of improvement has been shown to be influenced by psychological factors, coping strategies, and general health [32]. A prior study of individuals with knee OA showed incongruence between patient perception of functional ability and performance-based measurements [33]. However, this does not explain why there would be a difference between those in the upper and lower quartiles. Third, there may be limitations in the 6MWT. In persons with knee OA, 6MWT likely measures a balance of aerobic capacity, pain, musculoskeletal functional limitations, and psychosocial factors. The degree to which 6MWT varies based purely on OA-related functional limitations is unclear, and a ceiling effect in high-functioning individuals is plausible but unknown. Further research is required to better understand the construct of, and use of, 6MWT in individuals with knee OA across a range of disease severity, including baseline walking ability, and level of comorbidities.

This study adds to our understanding of MCID being a dynamic and context-specific entity [34]; instead of one value it may take on a range of values depending on relevant clinical characteristics. We found that those with worse baseline walking ability required greater change to be perceived as meaningful. The importance of baseline characteristics and clinical context to the MCID is underrepresented in OA literature, where generally a single value is sought. For example, for other commonly used measures, e.g. WOMAC, generally a single MCID is quoted [35]. By contrast, the chronic pain literature suggests that baseline level of pain is the most important factor to explain variability in MCID for pain across studies [20]. This should be considered in future research on OA outcome measures.

Our work had several strengths. To our knowledge, this is the first study to assess MCID for 6MWT by baseline level of walking ability, and importantly adds to the limited studies to date on 6MWT in persons with knee OA despite 6MWT being included in the OARSI-recommended set of performance-based measures [4]. We used an anchor-based approach linking to the patient perception of change to calculate the MCID. As indicated above, we dichotomized our sample as *improved* vs. *non-improved* at a relevant threshold for those undergoing TKA [29]. Limitations of our study include lack of specificity of our anchor question: participants were not asked *how* their walking was improved and this could be potentially relevant as 6MWT measures how much ground can be covered in 6 min. As discussed above, the anchor-based approach may be limited by several potential factors that influence perception of change, in addition to recall bias.

In conclusion, we provide an estimate of MCID for improvement for 6MWT in individuals with knee OA at 12 months after TKA for those in the lowest quartiles of baseline walking ability, that is, who walk ≤336 m at their pre-TKA 6MWT, and show that it is a dynamic entity. This study has raised several questions about the 6MWT and its use across the spectrum of OA disease severity. Given the importance of improvement in physical function for persons with knee OA, and the role of MCID in sample size calculation for clinical trials and interpreting study results, we suggest a better understanding of the construct of 6MWT is required including its use in persons with high vs low baseline levels of physical function.

## Data Availability

The datasets generated and/or analysed during the current study are not publicly available due to the Research Ethics Board-approved study protocol for the “BEST-Knee Study”. However, the data is available from the corresponding author on reasonable request.

## Abbreviations

6MWT: Six-Minute Walk Test
AUC: Area Under the Curve
BMI: Body Mass Index
IQR: Interquartile Range
KOOS: Knee injury and Osteoarthritis Outcome Score
MCID: Minimal Clinically Important Difference
OA: Osteoarthritis
OARSI: Osteoarthritis Research Society International
PHQ-8: Patient Health Questionnaire Depression Scale
ROC: Receiver-Operating Characteristic
SD: Standard Deviation
TKA: Total Knee Arthroplasty
WOMAC: Western Ontario and McMaster Universities Osteoarthritis Index
